# Clear cell renal carcinoma synchronous with dedifferentiated liposarcoma: a case report and review of the literature

**DOI:** 10.1186/s13256-019-2320-4

**Published:** 2020-01-08

**Authors:** Estefania Beltran, Juan Esteban Garcia-Robledo, Lisa X. Rodríguez-Rojas, Martin Rengifo, Bladimir Perez, Harry Pachajoa, Angela R. Zambrano

**Affiliations:** 1 Centro de Investigaciones Clínicas, Fundación Valle del Lili, Cali, Carrera 98 No. 18-49, Colombia; 2 Hematology/Oncology Department, Fundación Valle del Lili, Cali, Carrera 98 No. 18-49, Colombia; 3 Faculty of Health Sciences, Universidad Icesi, Cali, Calle 18 No. 122-135, Colombia; 4 Human Genetics Department, Fundación Valle del Lili, Cali, Carrera 98 No. 18-49, Colombia; 5 Diagnostic Imaging Department, Fundación Valle del Lili, Cali, Carrera 98 No. 18-49, Colombia; 6 Pathology Department, Fundación Valle del Lili, Cali, Carrera 98 No. 18-49, Colombia

**Keywords:** Multiple primary malignant neoplasms, Multiple primary tumors, Renal cell carcinoma, Liposarcoma, Next-generation sequencing

## Abstract

**Background:**

Multiple primary malignant neoplasms are not frequent but are increasing in incidence. Some of them are associated with genetic syndromes such as von Hippel-Lindau syndrome and Li-Fraumeni syndrome. Dedifferentiated liposarcoma is one of the rarest soft tissue tumors, and clear cell renal carcinoma is the most frequent kidney cancer. The concomitant presence of these tumors is extremely rare; however, some cases have been reported, none of them presenting with liposarcoma of the limbs. We report an interesting case of a patient with synchronous multiple primary tumors presenting with a very rare liposarcoma associated with renal cell carcinoma (a very rare association). A review of the literature and a collection of similar cases published previously are also provided.

**Case presentation:**

We report a case of a 62-year-old Hispanic man who presented to our institution with a left thigh mass compatible with dedifferentiated liposarcoma synchronous with metastatic clear cell renal carcinoma. Multiple treatment lines were provided with no response, with a further metastatic transformation. Genetic analysis by liquid biopsy showed some mutations that were not susceptible to targeted therapy. At the time of this report, the patient is undergoing palliative care because his nonresponsive metastatic disease persists.

**Conclusions:**

We present the first reported case of clear cell renal carcinoma synchronous with dedifferentiated liposarcoma of a limb. The association between renal cell carcinoma and dedifferentiated liposarcoma is unusual, and there are few reports of this presentation in the literature. More research about these tumors along with genetic tests needs to be performed to seek a better understanding of the fundamental basis of this rare association.

## Background

Multiple primary malignant neoplasms (MPMN) are rarely seen in clinical oncology practice [1]. Even though they are not frequent, their incidence is progressively increasing, mainly because of aging and the advancements in medical technology [2]. MPMN prevalence is estimated to be between 0.7% and 11.7% [3, 4]. MPMN can be synchronous when the second tumor presents in less than 6 months since the diagnosis of the first one, and metachronous if more than 6 months passes between diagnoses [5]. The most important risk factors related to MPMN are familial history of cancer, obesity, and smoking [6].

The presence of a malignant neoplasm has been shown to increase the risk of developing a second one in 20% of cases [7], and there are certain genetic conditions that might increase the risk of development of multiple tumors, such as von Hippel-Lindau (VHL) syndrome [8] and Li-Fraumeni syndrome [9].

Liposarcoma (LS) is the most common type of soft tissue tumor, representing approximately 20% of all sarcomas [10]. Dedifferentiated liposarcoma (DDLS) is a rare subtype of LS that has an aggressive behavior with high-risk histology and metastatic behavior; it accounts for a small percentage of all LS and is usually the consequence of progression from a well-differentiated LS [11].

According to the Global Cancer Observatory, during 2018, kidney cancer represented 2.2% of cancers worldwide, being the 16th most common cause of cancer and leading to approximately 1.8% of deaths related to cancer in the same year [12]. Eighty-five percent of kidney cancer cases were renal cell carcinoma (RCC), and the other 15% were renal pelvic carcinoma, a form of urothelial cancer [13]. Within RCC, clear cell renal cell carcinoma (ccRCC) is the most common type, accounting for 75–85% of RCC cases [14].

Chromosomal alterations are usually related with ccRCC [15]. The VHL tumor suppressor gene, located in chromosome 3p25.3, is frequently inactivated by deletion, microdeletion, or epigenetic mechanisms in approximately 80% of ccRCC cases [16, 17]. VHL is infrequently altered in other types of tumors [18]. Other chromosomal alterations involving translocations and deletions might also be seen in ccRCC [19].

In this article, we report a case of a Hispanic patient presenting with a very rare association of MPMN consisting of a metastatic RCC that was synchronous with DDLS. To our knowledge, this is the second case in the literature of this association and the first one in which LS presented in a limb. A review of the literature is also presented with a collection of similar cases reported previously.

## Case presentation

**Fig. 1:**
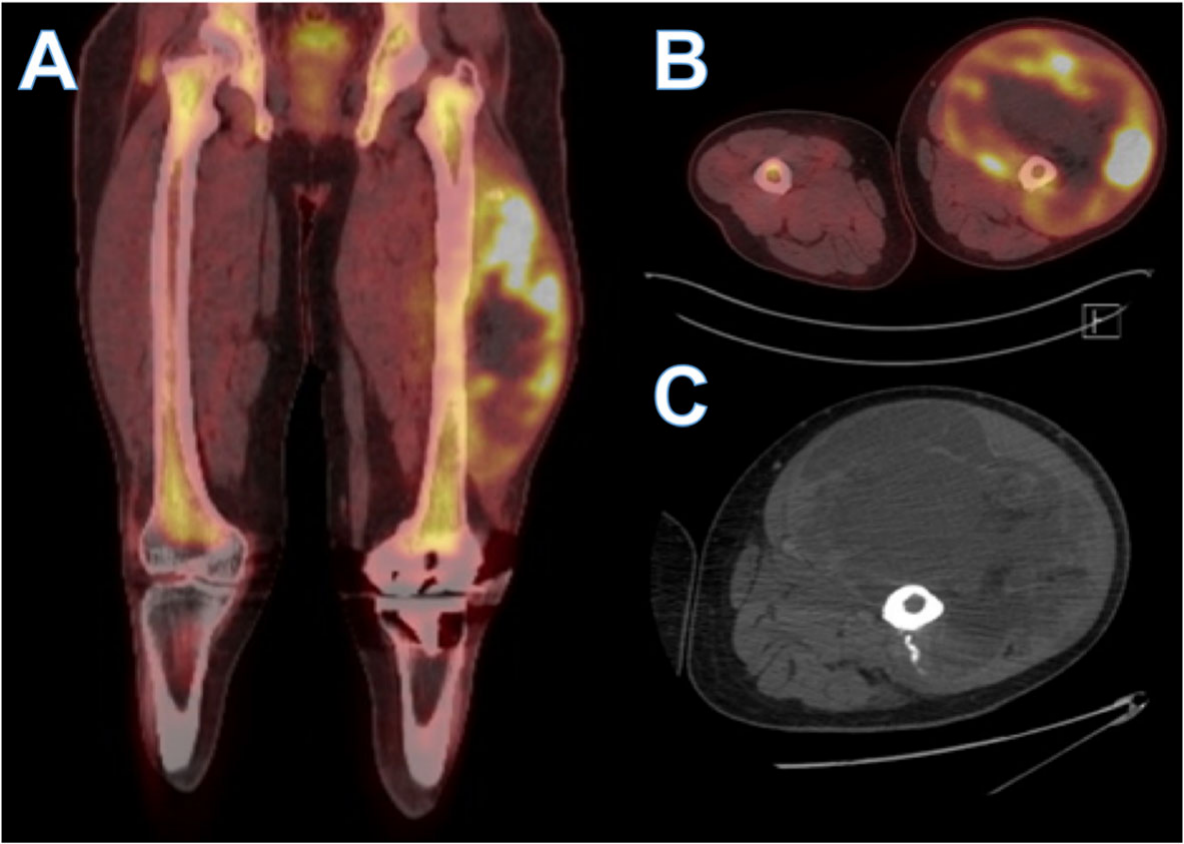
Positron emission tomography/computed tomography (PET-CT). **a** and **b** Lower limb PET-CT showing injury limited to the anterior compartment of the left thigh, which presents intense peripheral hypermetabolism. **c** Lower left limb CT showing injury limited to the anterior compartment with heterogeneous density of hypodense predominance

**Fig. 2:**
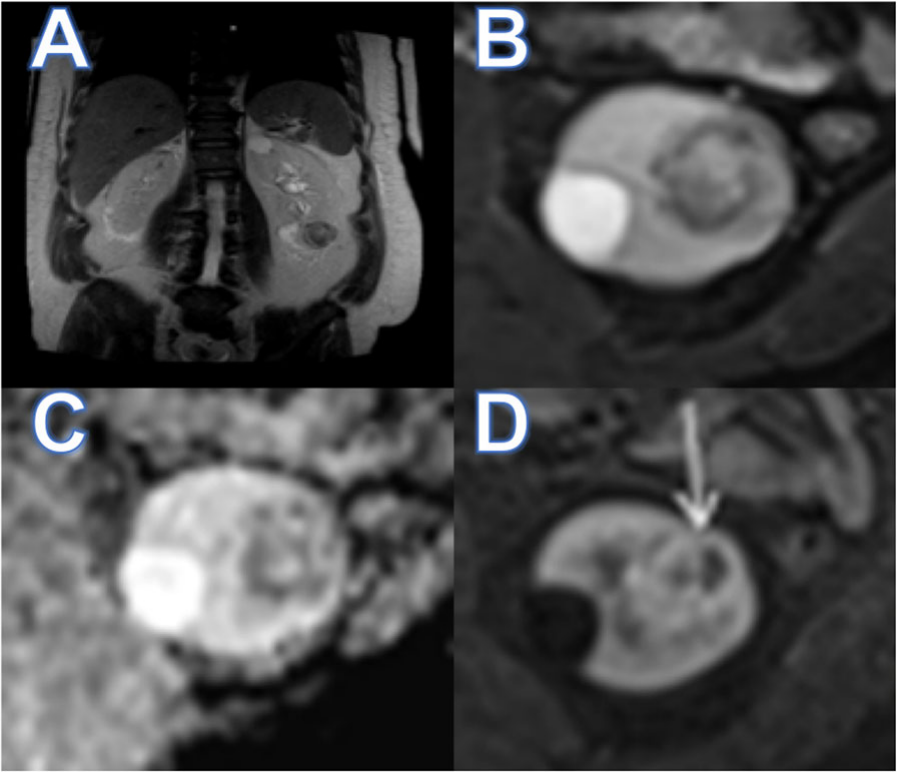
Abdominal magnetic resonance imaging. Left renal mass without extracapsular involvement. **a** T2 sequence. **b** 3D volumetric interpolated breath-hold sequence T2 fat-saturated + gadolinium enhanced image. **c** Apparent diffusion coefficient (ADC) map. **d** Axial T2 fat-saturated image (arrow). Injury of heterogeneous behavior with hypodense predominance in T2 sequences (**a** and **b**) Dissemination is being restricted with representation in the ADC maps (**c**) and irregular enhancement with contrast (**d**)

Our patient was a 62-year-old Hispanic African American man with a family history of four first-degree relatives with prostate cancer and past intervention of meniscus surgery 19 years ago with a flexion retraction sequela, but with no other remarkable history. He had started experiencing pain and noticed a mass appearance in the left thigh in February 2017. Six months later, he was seen as an outpatient in our orthopedic surgery unit. His physical examination revealed a large increase in volume in the middle third of the anterolateral aspect of the left thigh, but no signs of inflammation were found. A left thigh open biopsy of the lesion was done on September 2017, but the results only showed hemorrhage and fibrin deposits. Because the result was not conclusive, a new left thigh open biopsy was done 1 month after the first biopsy. The conclusion after immunohistochemistry was documented on December 20, 2017 (T = 0), as a fibrous tissue infiltrated by a neoplastic lesion of mesenchymal origin mixed with hemorrhage and tumor necrosis, high-grade pleomorphic sarcoma with giant cells randomly arranged, anisocytosis, anisokaryosis, and mitosis compatible with DDLS (T2bN1M0, stage III). Complementary workup done through December 2017 included esophagogastroduodenoscopy with a finding of noninvasive chronic antral gastritis, *Helicobacter pylori*-negative, and a colonoscopy that showed grade I internal hemorrhoids and mild diverticulosis. At the patient’s first clinical oncology appointment (T = 15 days), the physical examination evidenced good general health and weight of 95.2 kg. His vital signs were blood pressure 127/72 mmHg, pulse rate 109 beats/minute, respiratory rate 16 respirations/minute, oxygen saturation 98%, and no fever. His general physical examination was unremarkable except with regard to the left extremity, which showed a great neoplastic lesion with a circumference of 70 cm and a length of 37 cm associated with inguinal edema with the presence of peripheral pulses. That same day, the patient presented to the emergency room (ER) in order to start further workup and treatment. During his time in the ER, positron emission tomography/computed tomography (CT) (Fig. 1) was performed (T = 21 days), and the results showed a hypermetabolic neoplastic mass that compromised the proximal two-thirds of the left thigh with cortical changes and retroperitoneal and inguinal adenopathies, a nonhypermetabolic hypodense lesion in the left kidney, and a hypermetabolic node in the right thyroid lobe. Thyroid and urinary tract ultrasound scans (T = 23 days) exposed a pseudonodular image in the right thyroid lobe and a solid mass in the lower pole of the left kidney. Magnetic resonance imaging (MRI) of the abdomen revealed a mass on the inferior pole of the left kidney (Fig. 2) with dimensions of 7 × 2.9 × 3 cm, neoproliferative, without extracapsular or ganglionic involvement. Further studies included a percutaneous biopsy of the thyroid gland (T = 26 days) and a percutaneous CT-guided renal biopsy (T = 27 days), which showed a nonmalignant goiter and ccRCC as a new synchronic primary neoplasm (cT1aN0M0, stage I).

Treatment for LS was initiated with a first-line chemotherapy cocktail containing epirubicin (an antineoplastic agent belonging to the group of anthracyclines that intercalates between deoxyribonucleic acid (DNA) base pairs, inhibiting DNA replication and transcription) [20], ifosfamide (an antineoplastic agent belonging to the group of alkylating agents that acts in phase S of the cell cycle, interfering with DNA replication) [21], and mesna (a chemoprotective agent that reduce the risk of developing hemorrhagic cystitis after ifosfamide therapy) [22] (first cycle = 34 days; second cycle = 59 days; third cycle = 85 days). During treatment, the patient developed ifosfamide neurotoxicity manifested as myoclonus and altered state of consciousness that resolved without sequelae. An unfavorable evolution was seen for the sarcoma; however, stable disease was seen for the renal carcinoma. After first-line treatment, renal tumor size decreased considerably; yet, the sarcoma did not improve at all.

The treating physicians decided to perform radical surgery. A left hemipelvectomy was performed (T = 129 days). Second-line treatment for LS was initiated with a targeted therapy based on pazopanib, a tyrosine kinase inhibitor (T = 157 days). Despite good tolerance, a new pelvic and abdominal MRI (T = 439 days) evidenced infiltration of the iliac crest and left sacroiliac joint that infiltrated the iliopsoas muscle, left paraspinal muscle, and minor and middle left gluteal muscles. Chest, abdominal, and pelvic CT evidenced the presence of pulmonary metastases (T = 442 days); thus, a third line of treatment for LS with gemcitabine (an antineoplastic antimetabolite agent) and docetaxel (a taxane that interferes with microtubule dynamics) (first cycle = 445 days; second cycle = 467 days; third cycle = 493 days; fourth cycle = 516 days; fifth cycle = 537 days).

Another pelvic and abdominal MRI (T = 538 days) showed a nodular liver injury in segments VII and VIII. After chemotherapy, the patient received radiotherapy in the left thigh because of local tumor recurrence (T = 554 days). Finally, the patient was transferred to palliative care (T = 583 days) because there was no indication for more chemotherapy after three lines of treatment and persistent disease.

The last image obtained was a pelvic and abdominal MRI scan (T = 635 days) that visualized increased liver lesions, increased renal tumor mass, and increased pulmonary nodules; persistence of the iliac lesion; a new lesion in the right femur; and a new neoproliferative lesion in the right kidney.

**Fig. 3:**
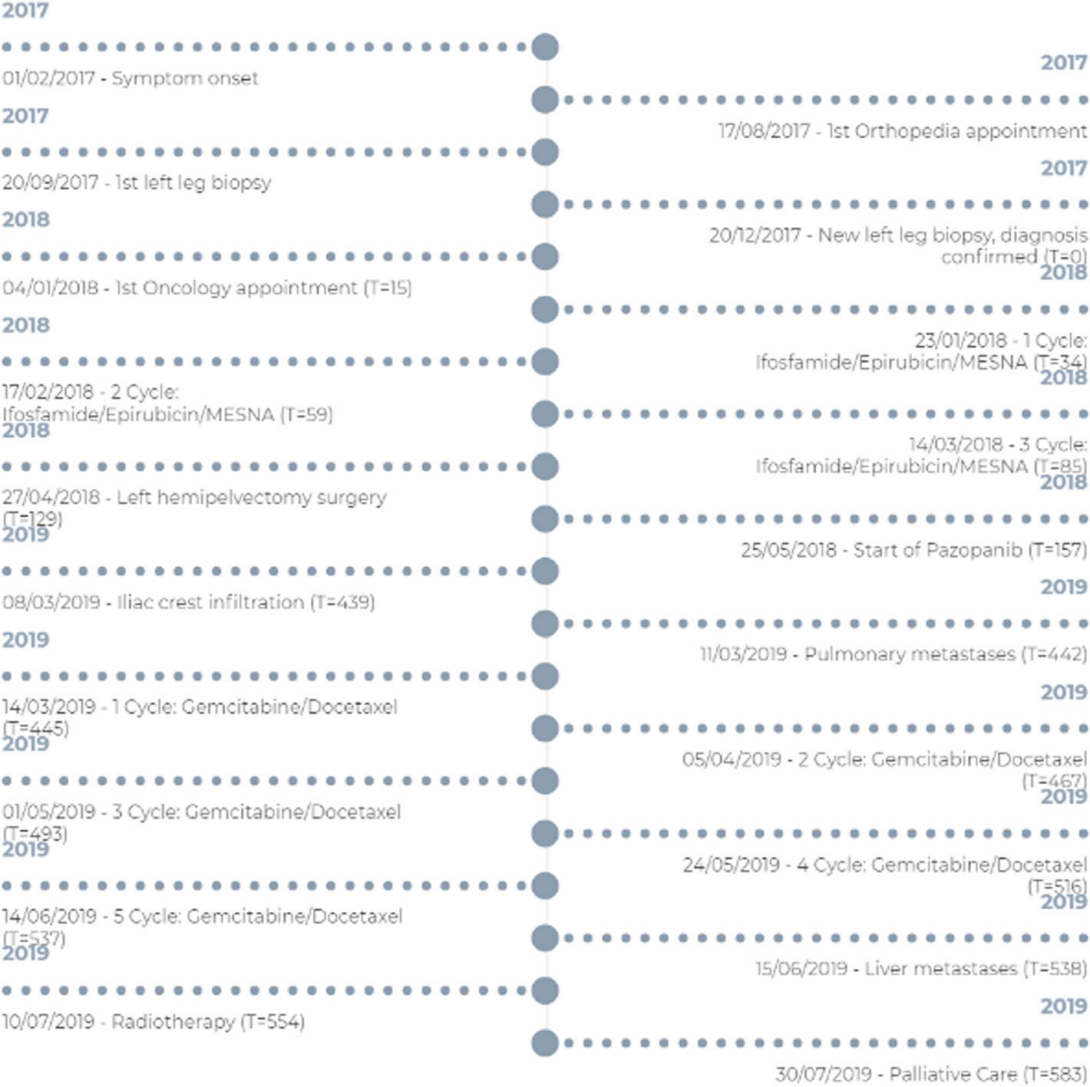
Timeline of clinical events

Once the kidney neoplasm was identified, the patient was evaluated in the genetics department, where a normal 46, XY karyotype was found. A somatic multigenic panel was performed by next-generation sequencing in circulating tumor DNA (liquid biopsy) to look for treatment alternatives. Four mutations were detected in *BRCA2*: L638FS*9, NF1: R1846FS*17, RB1: deletion exon 24, and TP53: R282W. None of these mutations has an action indicating a clinical benefit of these histological types of tumors (sarcoma and ccRCC). The instability state of microsatellites reported in the same test was indeterminate, and the result of germline genetic testing was negative. A summary timeline of all the remarkable events is provided in Fig. 3.

## Discussion

The incidence of RCC associated with other primary tumors varies between 12% and 30% of all RCC tumors. Second primary tumor locations, in order of frequency, are gastrointestinal, head and neck, uterine, and prostate. According to the literature, 90% of these secondary tumors are solid tumors [23], as in our patient’s case.

Males account for approximately 61% of RCC cases and for 63% of deaths related to it [23]. Patients with RCC have a threefold risk of developing a second primary tumor compared with other oncology patients in whom the first tumor is not an RCC.

We report a case of a patient with two synchronous primary malignant tumors (DDLS and left ccRCC). ccRCC was found as an incidentaloma with no symptoms of RCC. The patient’s karyotype was normal. Four pathogenic mutations were detected in the liquid biopsy, in the *BRCA2*, *NF1*, *RB1*, and *TP53* genes, which have not been related to a therapeutic benefit in terms of targeted therapy for these types of tumors. The patient was sent for genetic counseling for evaluation and to rule out hereditary cancer syndromes.

To our knowledge, this is the first report of ccRCC associated with DDLS from a limb. A total of six previous cases of RCC and LS have been reported, one of them a heart DDLS. All cases are summarized in Table 1.

**Table 1:** Summary of previously reported cases of patients with renal cell carcinoma concomitant with liposarcoma

Year of report	Age at diagnosis (years)	Sex	RCC histological type	RCC laterality	Location of liposarcoma	Reference
1994	71	Male	Granular cell	Right	Perirenal	Lewis *et al.* [24]
2003	58	Male	Papillary	Left	Cardiac	Gałazka *et al.* [25]
2008	79	Male	Papillary	Left	Retroperitoneum	Williamson *et al.* [26]
2009	60	Male	Clear cell	Right	Perirenal	Kinebuchi *et al.* [27]
2013	74	Male	Papillary	Left	Retroperitoneum	Frank *et al.* [28]
2014	34	Male	Clear cell	Left	Retroperitoneum	Hoshi *et al.* [29]
2014	55	Male	Clear cell	Left	Perirenal	Liu *et al.* [30]
2016	61	Female	Not mentioned	Right	Retroperitoneum	Reznichenko [31]

*RCC* renal cell carcinoma

RCCs have been seen to be associated with diverse second and even third primary tumors; however, their biological mechanisms are poorly understood. Some evidence shows a relationship between gastrointestinal stromal tumor and RCC, where genes coding for tyrosine kinase receptors such as c-MET and c-KIT might be involved, establishing the consideration of using tyrosine kinase inhibitors with the same target to treat them, but no other mechanisms have been proposed to date.

Our case is especially rare. DDLS only behaves as a metastatic disease in about 15% of cases [32]; most patients develop this tumor in the retroperitoneum, with very few cases developing in the limbs. Even though its etiology is unknown, it is related to some genetic alterations in the *MDM2*, *HMGA2*, and *CDK4* genes [33], which our patient did not have. Disease-specific 5-year overall survival (5yOS) is 44%; however, this is dependent on location, and some data show that patients with DDLS with limb lesions have a 5yOS > 90% [34].

We found some limitations manifested in the impossibility of finding a causal germline mutation that could explain the development of both tumors in our patient. Because of financial limitations of the patient, genetic analyses were deferred to some months after the initial diagnosis. No cultural limitations were present.

## Conclusions

Patients with RCC are at increased risk of developing a second primary solid malignancy. RCC associated with LS is a very rare presentation, and when evaluated by subtype, DDLS associated with RCC is very infrequent, with only two cases reported in the literature, including the present report.

## Data Availability

Not applicable.

## Abbreviations

5yOS: 5-year overall survival
ccRCC: Clear cell renal cell carcinoma
DDLS: Dedifferentiated liposarcoma
LS: Liposarcoma
MPMN: Multiple primary malignant neoplasms
MRI: Magnetic resonance imaging
PET/CT: Positron emission tomography/computed tomography
RCC: Renal cell carcinoma
VHL: Von Hippel-Lindau
